# Vaginal Cuff Dehiscence as an Unusual Cause of Pneumoperitoneum: A Case Series

**DOI:** 10.7759/cureus.106818

**Published:** 2026-04-10

**Authors:** Hania Ahmer, Hamdan Mallick, Nessreen Ghanem, Krishnaraj Mahendraraj, Theodoros Katsichtis

**Affiliations:** 1 Surgery, Bayhealth Hospital, Dover, USA

**Keywords:** bowel obstruction, pneumo peritoneum, small bowel herniation, vagina, vaginal cuff complications

## Abstract

Pneumoperitoneum is most commonly associated with gastrointestinal perforation and often prompts emergent surgical exploration; however, gynecologic sources such as vaginal cuff dehiscence (VCD) can rarely be the cause. We present two unusual cases of pneumoperitoneum secondary to VCD following remote hysterectomy, each with distinct presentations and operative findings. The first case involves a 38-year-old woman with prior Roux-en-Y gastric bypass and hysterectomy who presented with acute-on-chronic abdominal pain and imaging evidence of free intraperitoneal air. Diagnostic laparoscopy and intraoperative endoscopy ruled out gastrointestinal perforation, and subsequent exploration revealed complete VCD with a localized abscess and adherent small bowel requiring resection and open cuff repair reinforced with an omental patch. The second case involves an 83-year-old woman with a history of hysterectomy and planned vaginal prolapse repair who presented with suprapubic pain and vaginal bulging. Imaging revealed a small bowel herniation through the vaginal vault, and diagnostic laparoscopy confirmed complete cuff dehiscence, which was successfully repaired laparoscopically using barbed absorbable sutures. Both patients recovered uneventfully. VCD remains a rare but potentially serious postoperative complication, with a presentation as pneumoperitoneum being exceptionally uncommon. Awareness of this entity is critical to prevent unnecessary bowel resections when bowel viability is preserved and to guide timely, multidisciplinary management tailored to intraoperative findings.

## Introduction

Free intraperitoneal air is an alarming radiologic finding most commonly attributed to hollow viscus perforation and traditionally mandates emergent surgical evaluation. While gastrointestinal perforation accounts for the vast majority of cases, up to 10% of pneumoperitoneum is caused by nongastrointestinal sources such as thoracic, gynecologic, or iatrogenic etiologies [1]. One such cause, vaginal cuff dehiscence (VCD), is rare but clinically significant, particularly because it may present with evisceration of abdominal contents [2].

VCD occurs when the surgically closed vaginal vault separates following hysterectomy. Reported incidence ranges from 0.5% to 7.5%, with higher rates following laparoscopic and robotic hysterectomies due to increased thermal tissue injury, reduced tactile feedback, and higher intracorporeal energy use [2]. Several risk factors predispose patients to poor cuff healing, including endometriosis, prior pelvic surgery, atrophic vaginal mucosa, infection, radiation exposure, smoking, immunosuppression, and increased intra-abdominal pressure from chronic cough or straining [3]. The wide range of onset, from days to many years post-hysterectomy, adds further diagnostic complexity.

Typical manifestations of VCD include vaginal bleeding, watery discharge, pelvic pain, or overt evisceration of the bowel or omentum. However, atypical presentations are increasingly recognized. Case reports describe VCD presenting solely as pneumoperitoneum without gynecologic symptoms, sometimes decades after hysterectomy, leading to confusion with gastrointestinal perforation and unnecessary emergent laparotomy [4]. Because the clinical and radiographic findings can be nonspecific, the correct diagnosis often depends on a high index of suspicion, particularly in patients with a remote hysterectomy and no obvious gastrointestinal source.

In this case series, we present two patients who developed pneumoperitoneum secondary to VCD occurring 18 months and 20 years, respectively, after prior hysterectomy. Despite similar underlying pathology, their clinical presentations, operative findings, and optimal management strategies differed significantly. These cases underscore the importance of recognizing gynecologic causes of pneumoperitoneum and tailoring operative decision-making to individual circumstances.

## Case presentation

Case 1

A 38-year-old woman presented with a two-month history of intermittent abdominal pain, acutely worsening over three days. Her history included endometriosis, two cesarean sections with tubal ligation, a total abdominal hysterectomy 18 months earlier, and a Roux-en-Y gastric bypass two years prior.

She was afebrile with mild epigastric and pelvic tenderness. Laboratory values are shown in Table 1.

**Table 1:** Laboratory results.

Laboratory study	Laboratory result	Reference range
WBC	9,300 cells/µL	4,500-11,000 cells/µL
Hemoglobin	13.3 g/dL	13.5-17.5 g/dL
CRP	0.8 mg/dL	<1.0 mg/dL

CT abdomen and pelvis demonstrated free intraperitoneal air and pelvic fluid consistent with gastrointestinal perforation. Diagnostic laparoscopy revealed dense adhesions near the gastric pouch and Roux limb with no evidence of perforation. Intraoperative endoscopy confirmed intact anastomoses. Pelvic evaluation revealed purulent fluid and adhesions between the small bowel and the vaginal cuff.

**Figure 1:**
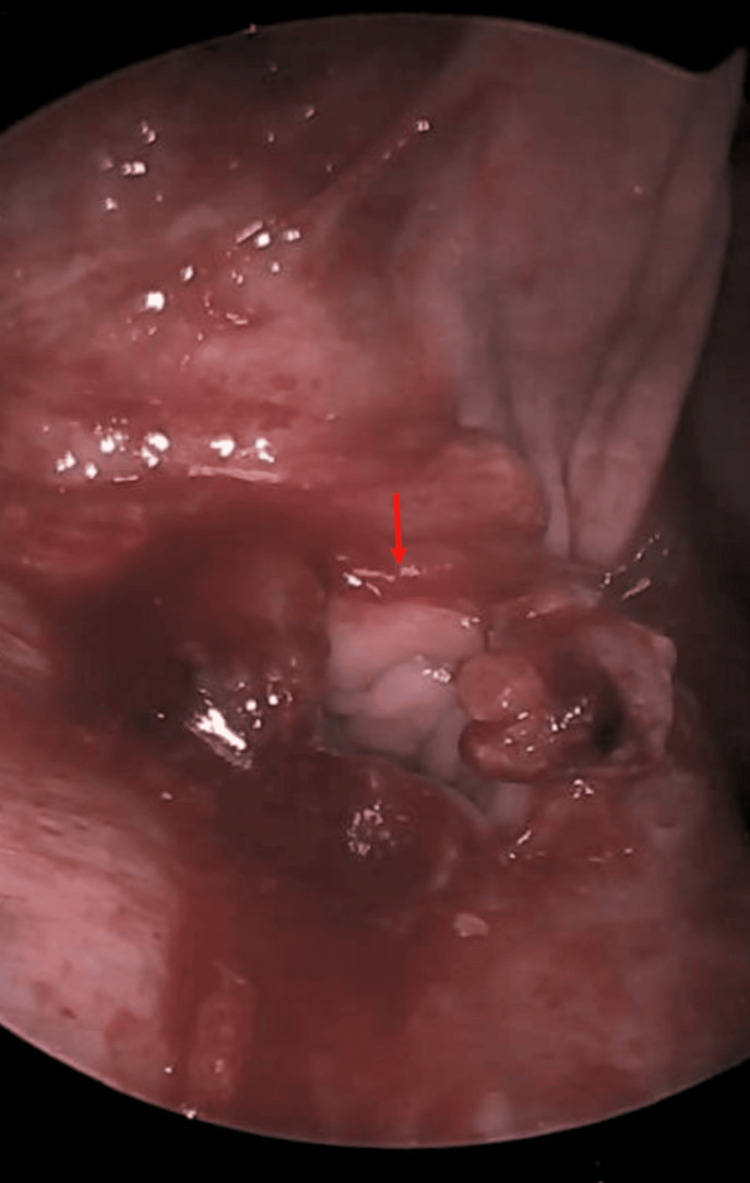
Open vaginal cuff seen on diagnostic laparoscopy. Red arrow: open vaginal cuff.

**Figure 2:**
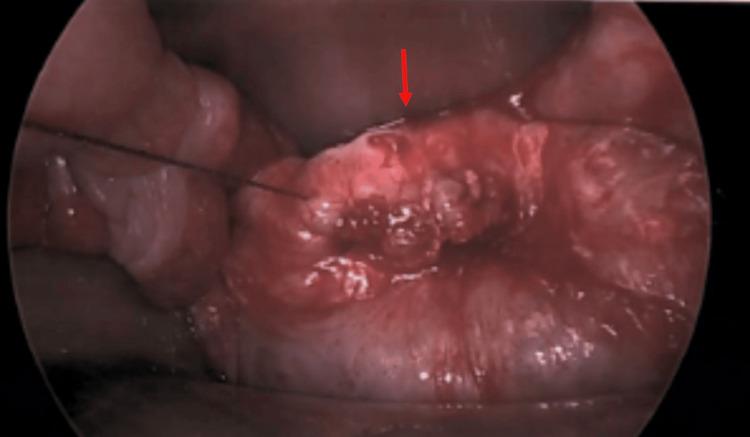
Vaginal cuff after suture closure red arrow), before omental patch reinforcement.

Diagnostic laparoscopy exposed a complete VCD with a localized abscess (Figure 1). Significant local inflammation and the presence of a small segment of ischemic bowel necessitated conversion to an open approach for a bowel resection and a thorough abdominal washout. The ischemic small bowel was a distal segment of the ileum. The adherent small bowel was resected with a primary stapled anastomosis. The vaginal cuff was repaired using barbed absorbable suture. The abdomen was reinsufflated, and the repair was assessed laparoscopically (Figure 2). A tongue of omentum was mobilized and laid over the repair to reinforce the closure. The patient recovered uneventfully and was discharged on postoperative day 3.

Case 2

**Figure 3:**
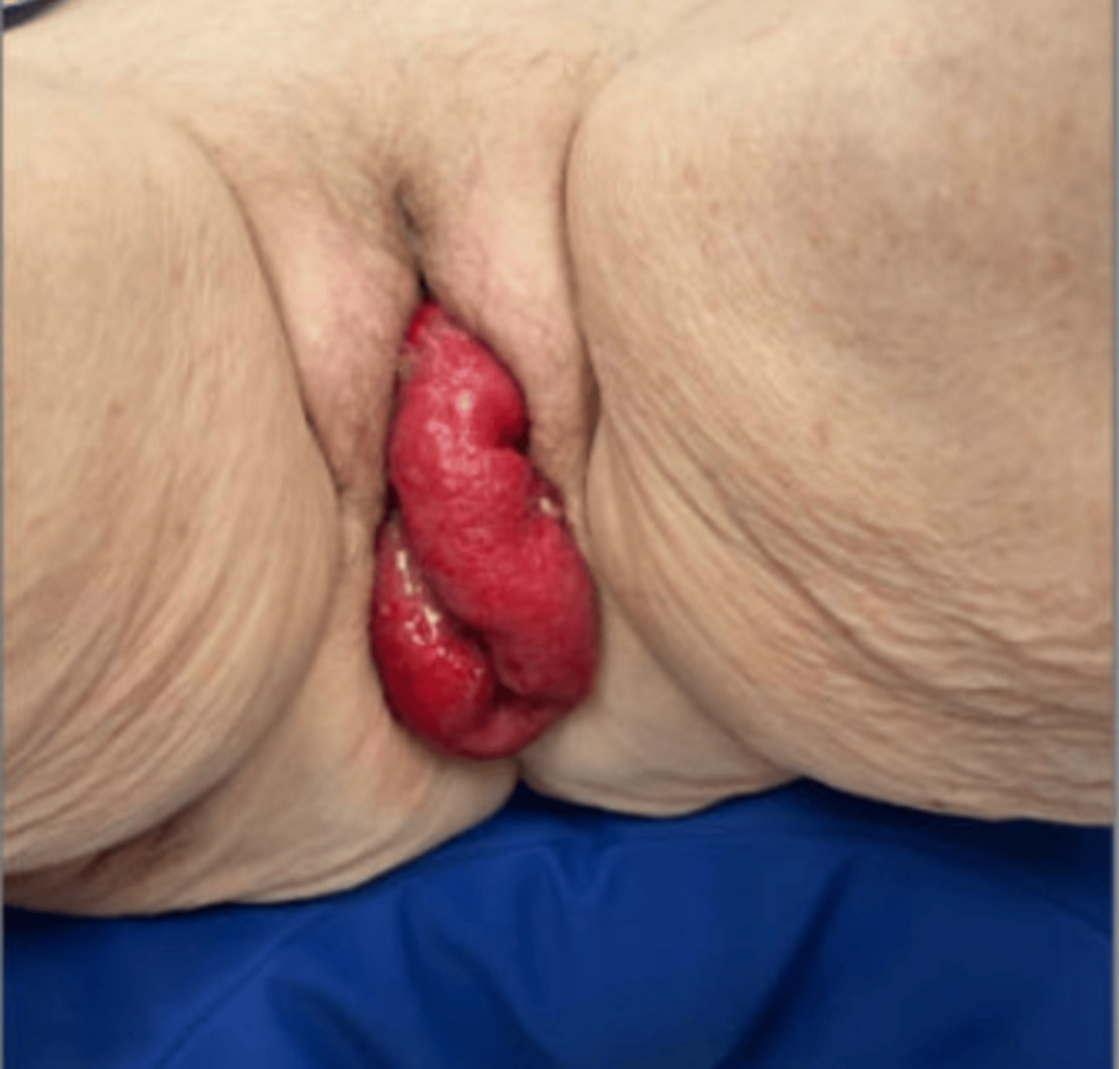
Transvaginal evisceration of the small bowel.

An 83-year-old woman with a history of total abdominal hysterectomy 20 years prior presented with suprapubic pain and vaginal bulging that began immediately after a bowel movement, approximately three hours before hospital presentation. Vital signs showed a blood pressure of 128/72 mmHg, temperature 37.1 °C, respiratory rate 18 breaths/min, pulse 96 beats/min, and oxygen saturation 98% on room air. The patient was being followed in the outpatient gynecology clinic and was scheduled for elective vaginal prolapse repair. Examination revealed eviscerated loops of hyperemic small bowel, protruding through the vaginal introitus (Figure 3).

**Figure 4:**
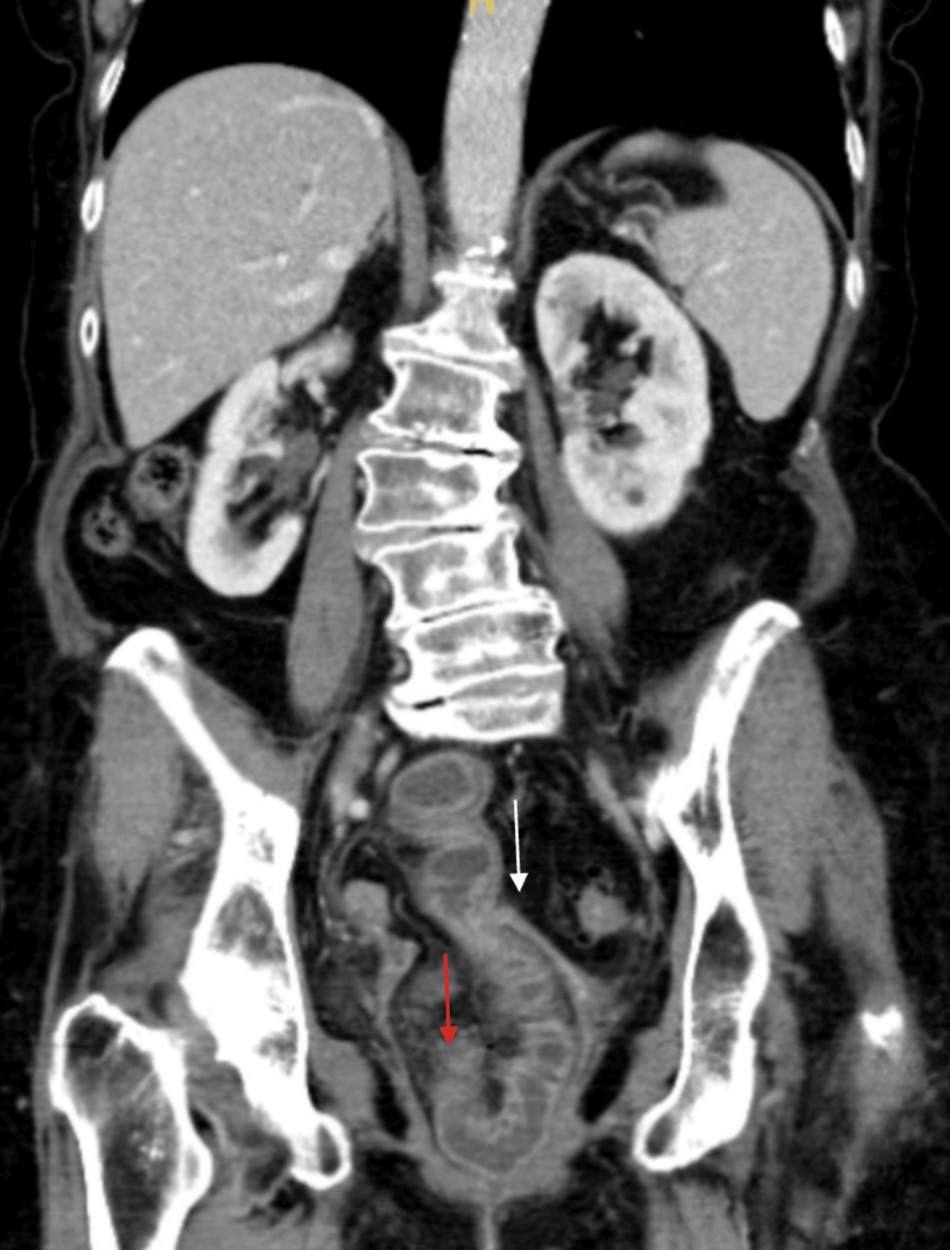
CT scan findings of vaginal cuff dehiscence. Red arrow: Dilated and thickened segment of the small bowel within the vaginal cuff. White arrow: Thickened and enlarged vaginal cuff.

Within an hour of reduction, she developed worsening abdominal pain with peritoneal signs. There was concern that the reduced bowel segment might have been nonviable or at risk for perforation. CT imaging demonstrated small bowel herniation through the vaginal vault with partial obstruction, pelvic free fluid, and trace pneumoperitoneum (Figure 4).

**Figure 5:**
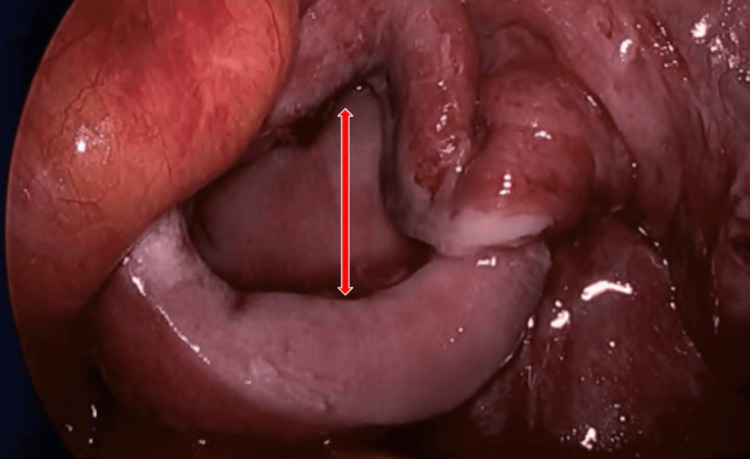
Vaginal cuff dehiscence demonstrating the longitudinal extent of the defect (red arrow).

**Figure 6:**
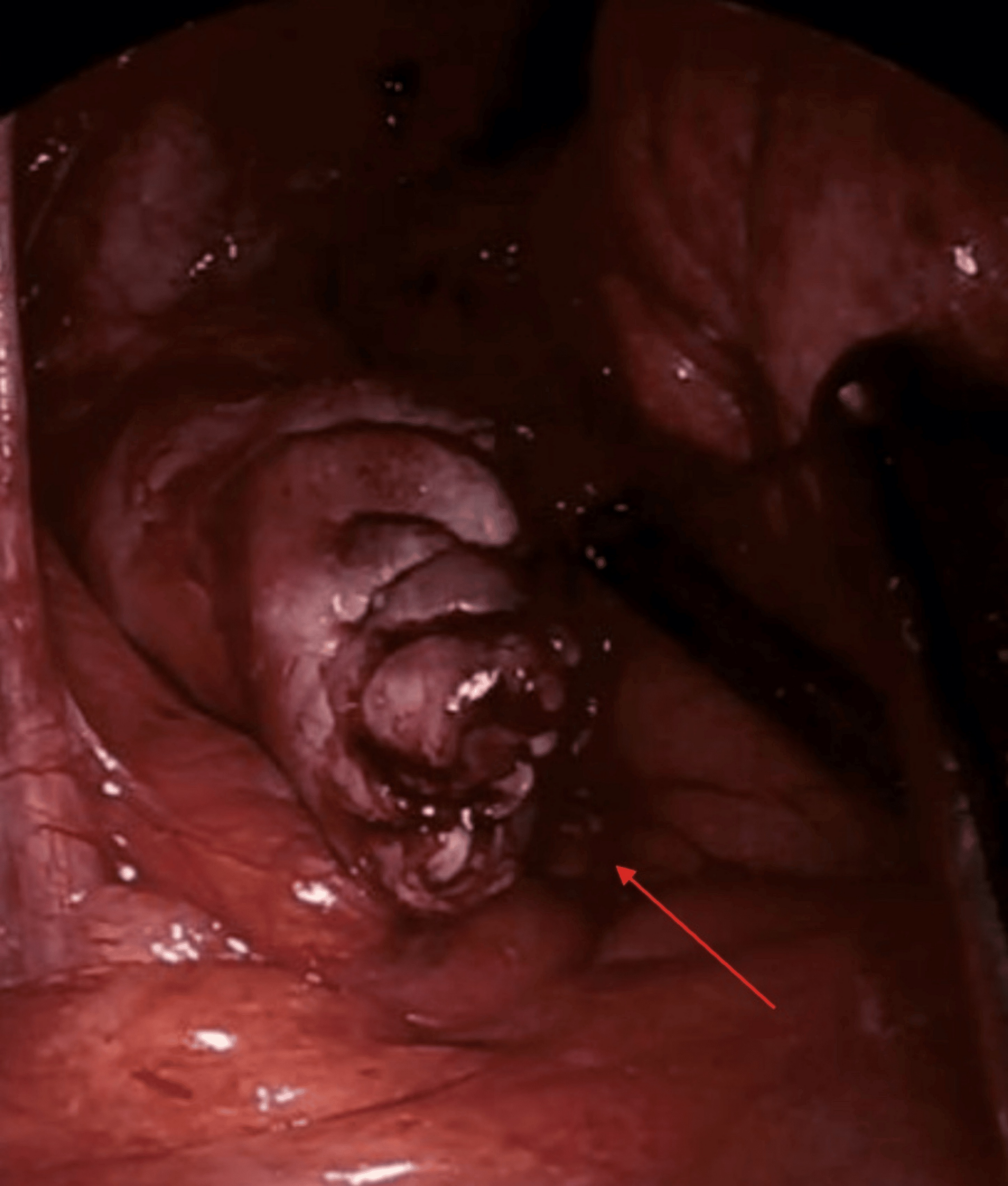
Vaginal cuff after suture repair. Red arrow: Repaired vaginal cuff.

Diagnostic laparoscopy revealed a giant vaginal cuff dehiscence with viable herniated bowel (Figure 5). The bowel was reduced and found to have preserved perfusion. The cuff was repaired laparoscopically in two layers using barbed absorbable suture (Figure 6). Her postoperative course was uncomplicated, and she was discharged with pelvic floor therapy follow-up.

## Discussion

VCD is a rare but potentially life-threatening complication following hysterectomy, with a reported incidence ranging from 0.5% to 7.5% depending on surgical approach [2]. Minimally invasive hysterectomy, particularly laparoscopic and robotic techniques, has consistently demonstrated higher rates of VCD compared with vaginal or open abdominal hysterectomy. This is believed to stem from factors such as increased use of monopolar electrocautery for colpotomy, which may impair vascularity and delay tissue healing [2,5]. Additional risk factors include smoking, chronic steroid use, prior radiation, infection, and conditions associated with poor tissue integrity, such as postmenopausal atrophy or endometriosis [3].

The clinical presentation of VCD varies widely, further complicating diagnosis. While classic symptoms include pelvic pain, vaginal bleeding, or evisceration of bowel or omentum, a subset of patients present atypically with abdominal pain and pneumoperitoneum in the absence of gynecologic complaints. This can create significant diagnostic confusion, particularly in emergency settings where free intraperitoneal air is strongly associated with gastrointestinal perforation and typically mandates urgent surgery [4,6]. Notably, past literature highlights that pneumoperitoneum can arise from numerous nonperforation etiologies, including postoperative retained air, thoracic sources, mechanical ventilation, sexual intercourse, and gynecologic pathology such as VCD [1]. In patients with remote hysterectomy, these alternative causes may be overlooked, especially when history is incomplete or symptoms are nonspecific. In our series, the patient in Case 1 specifically denied engaging in sexual intercourse in the eight weeks following her hysterectomy, reducing the likelihood of mechanically precipitated dehiscence. In contrast, the patient in Case 2, given the remote nature of her hysterectomy two decades earlier, was unable to reliably recall her immediate postoperative activity patterns, including the timing of resumption of intercourse.

The first case in our series underscores the diagnostic challenge: despite a history of hysterectomy, the patient’s pneumoperitoneum was interpreted as likely gastrointestinal in origin, prompting laparoscopy and subsequent conversion to laparotomy. Only after thorough inspection was the true source, a complete VCD complicated by abscess and adhesion-related small bowel involvement, identified. This scenario aligns with prior reports demonstrating that VCD can mimic a perforated viscus both clinically and radiographically [7].

In contrast, our second patient demonstrated classic evisceration physiology, with small bowel prolapse through the vaginal vault. Her presentation highlights a different end of the VCD spectrum in which herniation occurs without ischemia, allowing for minimally invasive repair. The favorable outcome in this case aligns with reports suggesting that early reduction and laparoscopic cuff repair can be safe and effective when bowel viability is preserved [2,7].

Management strategies for VCD depend on patient stability, extent of evisceration, and presence of contamination or bowel injury. Diagnostic laparoscopy is often the preferred initial approach in stable patients, offering the ability to evaluate bowel viability, reduce herniation, and repair the cuff. However, dense adhesions, unclear anatomy, or suspected perforation may necessitate conversion to open exploration, as occurred in our first case. Repair is typically performed with delayed absorbable or barbed sutures in one or two layers, with some authors recommending omental interposition to reinforce healing in cases with significant inflammation or tissue compromise [7].

Complications of VCD can lead to bowel evisceration, which can result in necrosis, perforation, and peritonitis [3]. Prevention strategies during hysterectomy emphasize minimizing thermal injury, ensuring adequate tissue perfusion, and closing the cuff with strong, evenly spaced sutures that incorporate the full thickness of the vaginal wall. Postoperatively, restricting intercourse, lifting, and activities that raise intra-abdominal pressure is recommended for at least 6 weeks by most authors [8].

## Conclusions

VCD is a rare but important cause of pneumoperitoneum and should be considered in post-hysterectomy patients presenting with unexplained free air. Early recognition may help avoid unnecessary bowel resections and facilitate definitive repair when bowel viability is preserved. Collaboration between general and gynecologic surgeons may optimize outcomes, particularly in complex or delayed presentations.
